# Performance of an innovative culture-based digital dipstick for detection of bacteriuria

**DOI:** 10.1128/spectrum.03613-23

**Published:** 2023-12-13

**Authors:** Emre Iseri, Sara Nilsson, Alex van Belkum, Wouter van der Wijngaart, Volkan Özenci

**Affiliations:** 1 Division of Micro and Nanosystems, KTH Royal Institute of Technology, Stockholm, Sweden; 2 UTIlizer AB, Stockholm, Sweden; 3 Department of Clinical Microbiology, Karolinska University Hospital, Stockholm, Sweden; 4 BaseClear BV, Leiden, the Netherlands; 5 Department of Laboratory Medicine, Division of Clinical Microbiology, Karolinska Institutet, Stockholm, Sweden; Johns Hopkins Hospital, Baltimore, Maryland, USA

**Keywords:** point of care, *in vitro* diagnostics, urinary tract infection, microbiological culture, bacteriuria, dipstick

## Abstract

UTI-lizer is a recent digital format for easy-to-use culture-based detection, preliminary identification, and quantification of bacteria in urine at the point of care (PoC). This study aimed to evaluate the diagnostic accuracy of UTI-lizer tests for detection of bacteriuria caused by five common bacterial species: *Escherichia coli*, *Klebsiella pneumoniae*, *Enterococcus faecalis*, *Proteus mirabilis*, and *Staphylococcus saprophyticus*. We evaluated the accuracy of UTI-lizer tests by comparing test results of UTI-lizer with those of current standard bacterial culture-based diagnostics in clinical microbiology laboratories in a retrospective and a prospective study. Comparator methods were classical bacterial culture in combination with matrix-assisted laser desorption/ionization-time of flight mass spectrometry mediated bacterial identification. In the retrospective study, we tested 104 urine samples with in-panel microorganisms, plain urethral microbiota, and culture-negative samples. In the prospective study, we used 137 urine samples within 10 hours of their collection at general practitioner clinics. The retrospective study demonstrated 100% sensitivity and specificity in the detection of bacteriuria, and 98.6% sensitivity and 96.8% specificity in identifying primary pathogens with UTI-lizer when compared to clinical standards. *S. saprophyticus* and *E. coli* could not be distinguished. The combined nitrite and esterase test predicted the presence of bacteriuria in only 36.5% of cases. The prospective study demonstrated 100% sensitivity and 89.6% specificity in the detection of significant bacteriuria for in-panel microorganisms with a coverage rate of 88.3% (121/137). This study indicates that digital dipsticks are a promising alternative for the detection of the five main pathogens that cause the vast majority of urinary tract infections (UTIs). The results demonstrate that digital dipsticks have the potential to uniquely provide—in primary care or at the point of care—a UTI diagnostic quality on par with that of current gold-standard testing. The ease of testing, rapid test handling time, and time to result as well as simple test equipment make digital dipsticks an attractive solution for decentralized testing for bacteriuria and with that improvement in UTI diagnostics. These results motivate future studies to validate the use of UTI-lizer at the PoC setting.

**IMPORTANCE:**

In this study, we explore the transformative potential of UTI-lizer, an emerging technology not yet commercially available. Our manuscript shows that UTI-lizer is a promising alternative for detecting the five main pathogens that cause urinary tract infections (UTIs). The results also indicate that digital dipsticks have the potential to uniquely provide UTI diagnostic quality on par with that of gold-standard testing, with the added benefits of ease of testing, rapid test handling time, and simple test equipment. This technology can be helpful in quickly ruling out bacterial infections and reducing the unnecessary use of antibiotics, especially in primary care settings or at the point of care. Moreover, the UTI-lizer test can reduce the number of negative urine samples sent to central laboratories, thus easing the burden of UTI diagnostics on the healthcare system. We believe our study, as well as current and upcoming research based on this technology, is highly relevant for clinical microbiologists, microbiology scientists, general practitioners, and urologists.

## INTRODUCTION

Urinary tract infections (UTIs) are among the most common bacterial infections physicians encounter (1). More than 50% of women are affected by UTIs at least once in their lifetime, and, therefore, most are treated with antibiotics at least once by the age of 24 (2, 3). In primary care, practitioners often choose to treat their patients empirically by antibiotics, immediately after the clinical examination, even before urine culture confirms the appropriateness of the antibiotic provided. Moreover, in most countries, diagnostic tests are not even requested to substantiate the empirical antibiotic treatment since these tests cost more than the treatment itself (4 – 7). These treatment strategies for UTIs in primary care, elderly care, or at the point of care (PoC) may lead to wrong or delayed treatments and misuse of antibiotics (6, 8). This increases the risk for morbidity including chronic or recurrent UTIs and developing antimicrobial resistance (8, 9). Yet, many guidelines still favor the conventional treatment strategy simply because no current PoC diagnostic test can improve the management of UTIs without burdening healthcare systems.

Today’s gold-standard diagnostic method is urine culturing. This phenotypic test takes around 24–48 hours and needs trained personnel and central laboratory facilities (5). Therefore, urine samples need to be transferred to central laboratories for testing, resulting in a loss of time for the patient and complexities for the healthcare personnel in primary care. Dipslides are alternative culture-based tests that offer faster (16–24 hours instead of 24–48 hours) and cheaper diagnoses in general practice (10). However, when used in daily practice, studies show that the sensitivity and diagnostic precision of these devices are poor compared to routine microbiological culturing (11, 12). Therefore, their use in UTI diagnosis remains limited. Another alternative culture-based test is Flexicult, which decreases the culturing test time by running antibiotic susceptibility tests (AST) parallel in the different compartments of the same culture plate (13). Tests offer clinically relevant results. However, they do not offer a cost-effective solution as this format requires considerable hands-on time and expertise for preparation and evaluation (6).

The most common pathogen in acute uncomplicated UTIs *is Escherichia coli* (*E. coli*), accounting for 80% of all cases (14). Other pathogens such as *Klebsiella pneumoniae (K. pneumoniae*), *Enterococcus faecalis* (*E. faecalis*), *Proteus mirabilis* (*P. mirabilis*), and *Staphylococcus saprophyticus* (*S. saprophyticus*) are infrequent causes of uncomplicated UTIs but still considered to be relevant pathogens. The frequency and antibiotic resistance patterns of these species may vary with geographical location. Therefore, quantifying these pathogens is crucial for accurate diagnosis and appropriate treatment of UTIs. A test that can accurately identify and quantify these species is essential for successfully managing urinary tract infections.

In primary care and other PoC settings such as long-term care or pregnancy and maternity care, urinary dipsticks (nitrite and leukocyte esterase strips) are used to confirm infection; however, they have a limited value in improving diagnostics (15 – 17) and are, therefore, only considered for screening purposes. Antibody-based lateral flow immunoassays provide an alternative screening method (18). Although these tests may offer yes/no answers for significant bacteriuria for screening purposes, their validity remains unproven for the intended use cases.

Direct pathogen detection and identification from urine with mass spectroscopy (19), PCR (20, 21), and flow cytometry (22) have been previously considered for improving UTI diagnostics. In recent years, single-cell imaging-based phenotypic AST (23) and loop-mediated isothermal amplification-based tests (24) have been demonstrated for UTI diagnosis in proof-of-concept studies. All these methods promise rapid turnaround time as well as high sensitivity and specificity. However, these methods are restricted to use in well-equipped clinical microbiology laboratories since they all feature one or more entry barriers for use at the PoC: expensive test equipment need, expensive unit test (disposable), trained operator need, extensive sample preparation steps (expert time need).

Despite previous efforts to improve UTI management, the need for more rapid, clinically relevant, and cost-effective tests for the PoC diagnosis of UTIs remains. Recently, a new culture-based method, UTI-lizer (UTIlizer AB, Stockholm, Sweden), also known as digital dipstick, was introduced (25). This emerging technology, which is not yet commercially available, demonstrated miniaturized bacterial detection and digital quantitation for PoC use. UTI-lizer miniaturizes culturing into a digital bioassay format. Additionally, minimal handling time is needed to initialize the test (<1 minute), and complicated external equipment is not required. In other words, the test can be facilitated at primary care or even PoC without pre-investment or a need for highly trained personnel. Here, we evaluate the performance of UTI-lizer for the detection of five common UTI pathogens, *E. coli*, *K. pneumoniae*, *E. faecalis*, *P. mirabilis*, and *S. saprophyticus* (from here onward “panel microorganisms”), in urine samples (retrospective) and assess the overall performance as a bacteriuria diagnostic test on another set of urine samples (prospective).

## MATERIALS AND METHODS

### Experimental setting and sample collection

The study was performed between March 2022 and May 2022 at the Department of Clinical Microbiology at Karolinska University Hospital in Huddinge, Sweden.

For the retrospective study, urine samples with a minimum volume of 5 mL, which had been stored at 2–8℃ for a maximum of 5 days, were selected for use. To evaluate all five panel microorganisms, primarily monomicrobial urine samples (see Supplementary Information 3) were used for this study due to the relatively low occurrence of the pathogens except *E. coli*. These samples were processed using standard urine culturing followed by matrix-assisted laser desorption/ionization-time of flight mass spectrometry (MALDI-TOF MS) described below and Supplementary Information 1. Patient urine samples were stored for a maximum of 5 days at 2–8°C according to local clinical guidelines after standard urine culturing. There were no other selection criteria applied for this part of the study.

For the prospective study, clinical urine samples from 53 different general practitioner offices in Stockholm were collected throughout the study. To ensure sample selection randomness, urine samples were included in the study based on submission order, ranging from 6 to 24 with a minimum volume of 5 mL. Consequently, the number of samples tested each day varied. Number of patients in different age and gender groups included in prospective study is summarized in Table 1.

**TABLE 1:** Age and gender information of patients included in prospective study (*n* = 121)

Age	Male	Female	Total
0–14	3	9	12
15–47	10	24	34
48–63	6	14	20
64+	19	36	55
Median (IQR^*a*^)	63.5 (36.25–76)	58 (35–75)	59 (35–75)
Average	54.6	53.8	54.1

^*a*^ Interquartile range.

### Standard urine culturing with agar plates and MALDI-TOF MS

Mid-stream urine samples were cultured on blood and chromogenic Brilliance UTI Agar (Thermo Fischer Scientific, Basingstoke, UK) by laboratory personnel using the automatic BD Kiestra InoqulA Sample Processor (BD Kiestra B.V., Drachten, The Netherlands) or manually according to local clinical guidelines. All agar plates were prepared by the Substrate Department of Karolinska University Hospital in Huddinge, Sweden. They were incubated at 35–37°C for 16–24 hours, and laboratory personnel evaluated and confirmed microbiological findings from agar plates by using MALDI-TOF MS (Bruker Daltonics GmbH, Bremen, Germany) (Supplementary Information 1).

### Nitrite and leukocyte esterase testing with urine strips

Multistix 7 (Siemens, Erlangen, Germany) were used and read according to the manufacturer’s instructions. The nitrite and leukocyte esterase fields were subjectively read and compared to the color scale after 60 and 120 seconds.

### Urine culturing with UTI-lizer

UTI-lizer with incubation boxes and user instructions were provided by UTIlizer AB, Stockholm, Sweden. The UTI-lizer dipsticks, a.k.a. digital dipsticks, were produced according to the description provided in previous studies (25, 26). These dipsticks were made to offer a simple way on sample inoculation and “digital” readout by untrained users. In this context, “digital” means that the sample is divided into numerous isolated microreactors, with most of them containing either 0 or 1 target analyte particle. Subsequently, an assay conducted in each microreactor quantitatively and discretely detects the presence of the analyte. Furthermore, aside from its user-friendliness and automated quantification process, this format provides significant multiplexing capabilities, as elaborated upon in the discussion section of the paper. These capabilities have the potential to address the encountered issues and enhance diagnostic quality.

**Fig 1:**
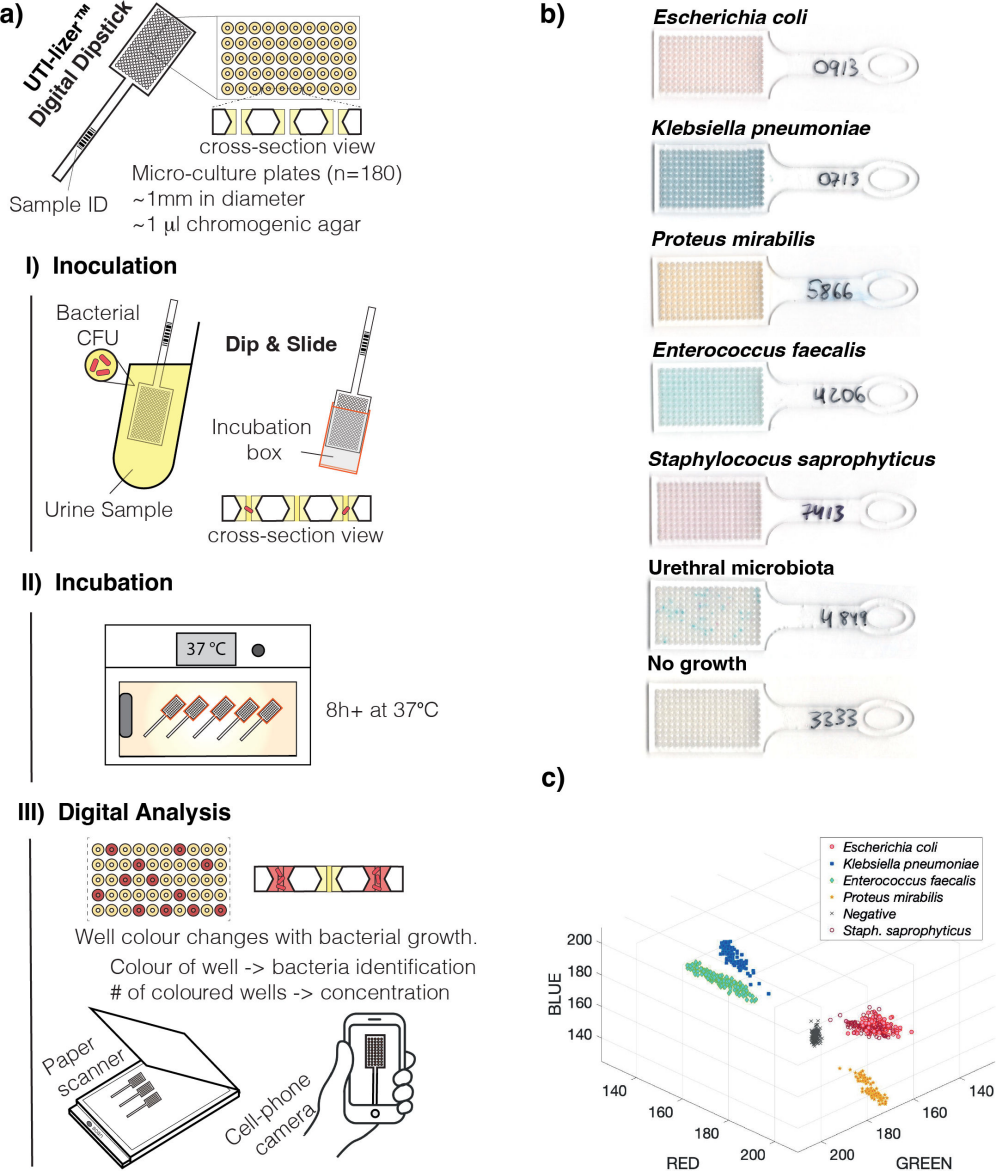
Urine culturing with UTI-lizer. (a) Digital dipsticks contain 180 microculture wells and are operated in three steps: dip-and-slide inoculation, incubation, and digital image analysis using a cellphone or scanner. (b) Photographs of digital dipsticks after operation on clinical urine samples (25). (c) Red-green-blue (RGB) color plot, where every dot is the color of one individual well of a digital dipstick after culture of urine samples (results for five positive and one negative sample are here combined, where different colors indicate different samples).

The operation steps of digital dipsticks are illustrated in Fig. 1. It involves immersing a dipstick in raw urine, to inoculate small amount of urine in each well. After placing the dipstick in a small incubation box, it is incubated at 37°C. Bacterial colonies induce a color change in positive wells, while negative wells remain uncolored. This color difference is used to count and quantify bacterial concentration, and the specific colors identify the bacterial species by image processing. Overall hands-on time of the UTI-lizer is around 1–2 minutes.

Digital dipsticks were dipped in stirred urine for the capture of sample in the wells and then placed in a mini-incubation box. Then, the device was placed in a humid incubator at 36°C. To align this study to the laboratory workflow of Karolinska University Hospital, all incubations were performed overnight (~12–15 hours). The readout images were taken using a color paper scanner Perfection V39 (Epson). Example images are shown in Fig. 1b. Additionally, some samples were photographed with Iphone 8S for further testing. All images were uploaded on the server of UTIlizer AB for image processing and readout of results. In this study, images obtained from the paper scanner are used for the final evaluation of UTI-lizer. Additionally, evaluation based on cellphone images of dipsticks and evaluation of information obtained by the naked eye were also performed. Additional comparisons and example images can be found in Supplementary Information 2.

### Performance analysis on the panel pathogens

The performance of UTI-lizer for detecting the panel microorganism was analyzed *ex vivo* in a non-consecutive case series. All samples were processed and evaluated by laboratory personnel prior to testing.

In the first part (retrospective), 74 predominantly monomicrobial urine samples [*E. coli* (*n* = 29), *K. pneumoniae* (*n* = 15), *E. faecalis* (*n* = 10), *P. mirabilis* (*n* = 10), and *S. saprophyticus* (*n* = 10)] and 20 urine samples with natural urethral microbiota were collected. Five ATCC strains (*E. coli* ATCC 25922, *K. pneumoniae* ATCC 25955, *E. faecalis* ATCC 29212, *P. mirabilis* ATCC 29245, and *S. saprophyticus* ATCC 15305) were separately inoculated in 0.9% NaCl to a turbidity of 0.50 McFarland and then diluted in negative urine until a final concentration of about 5 × 10^5^ CFU/mL was obtained as positive controls. Ten negative urine samples without visible growth on agar were used as negative controls. One urine sample per patient was tested simultaneously with UTI-lizer, standard urine culture (gold standard), and Multistix 7 according to the manufacturer’s instructions.

In the second part of the study (prospective), the performance of UTI-lizer was evaluated on prospective clinical samples from 53 different healthcare clinics in Stockholm and all experiments were performed in Karolinska Hospital Clinical Microbiology Laboratory in Huddinge. Samples (*n* = 137) were tested with UTI-lizer within the first 10 hours of collection through the workflow. Since *S. saprophyticus* growth results in a similar red/pink color change as *E. coli* growth, all red/pink color changes on the dipstick are defined as *E. coli* based on the general high prevalence of these bacteria.

**Fig 2:**
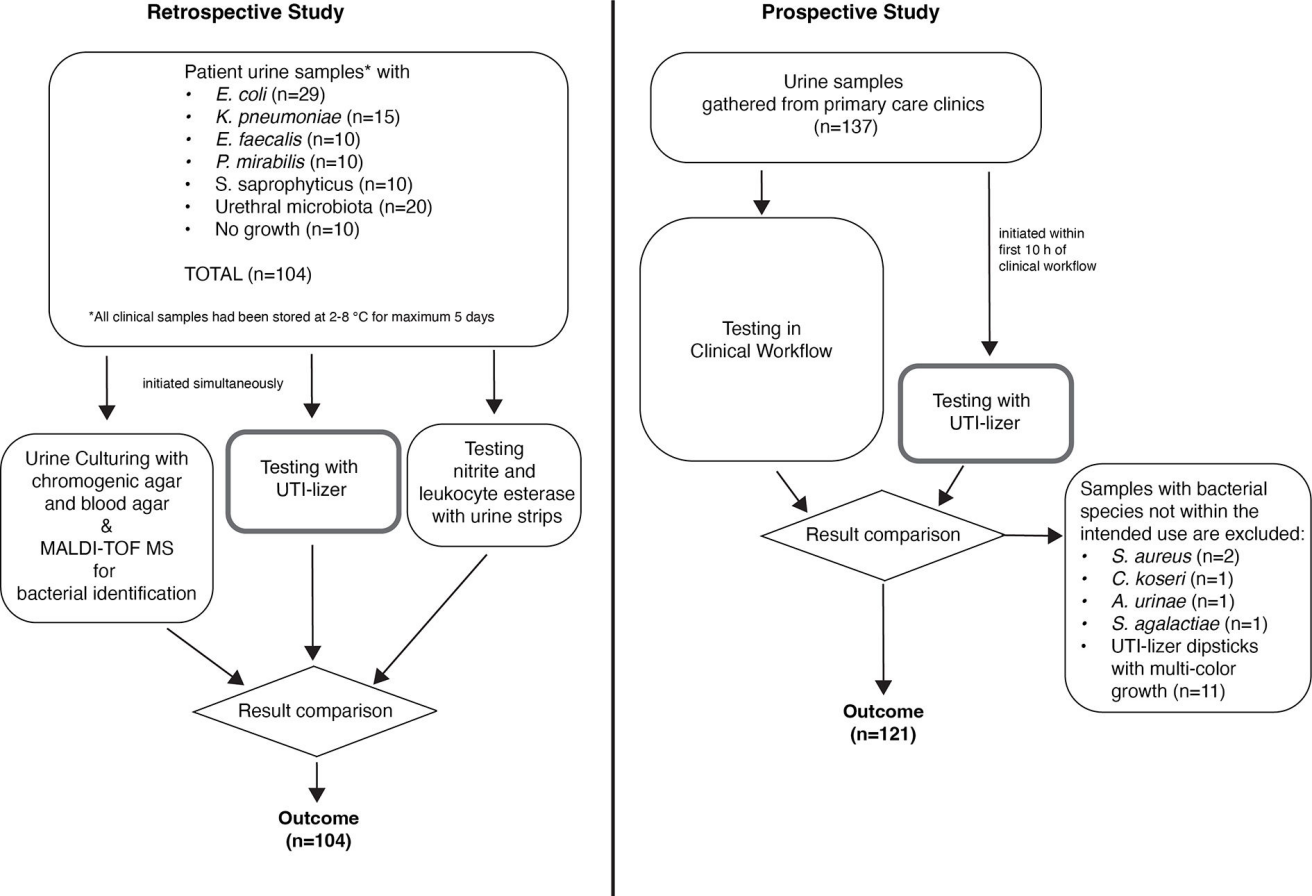
Flowcharts of retrospective and prospective studies.

The retrospective (first part) and prospective (second part) study flowcharts are shown in Fig. 2.

## RESULTS

In the first part of the study (retrospective), UTI-lizer demonstrated 100% sensitivity and specificity in detecting bacteriuria, and 98.6% sensitivity and 96.8% specificity in identifying the five microorganisms when results are compared to classical cultivation completed by MALDI-TOF MS (*n* = 104). Additionally, results from nitrite, leukocyte esterase, and combined nitrite and leukocyte esterase tests were able to determine 43.2%, 79.7%, and 36.5% of bacteriuria, respectively. The results of the retrospective study are summarized in Table 2.

**TABLE 2:** Retrospective study results: performance of UTI-lizer in determining bacteriuria and identifying specific microorganisms^*a*^ when compared to standard urine culture and MALDI-TOF MS and additional comparison with nitrite and leukocyte esterase test

Identification	No. of true/false positives	No. of true/false negatives	Sensitivity (%)	Specificity (%)	Positive predictive value (%)	Negative predictive value (%)	Nitrite positive/negative	Leukocyte esterase postive/negative	Nitrite and leukocyte esterase positive/negative
Bacteriuriadetermination **^*b*^**	74/0	30/0	100	100	100	100	N.A.	N.A.	N.A.
Microorganism identification **^*c*^**									
Total	73/1	30/1	98.6	96.8	98.6	96.8	N.A.	N.A.	N.A.
Gram-negativebacteria ID									
Total	53/0	50/1	98.2	100	100	98	30/24	43/11	25/29
*Escherichia coli*	29/0	75/0	100	100	100	100	20/9	25/4	16/13
*Klebsiella pneumoniae*	14/0	88/1	93.3	100	100	98.9	3/12	9/6	3/12
*Proteus mirabilis*	10/0	94/0	100	100	100	100	7/3	9/1	6/4
Gram-positivebacteria ID									
Total	20/1	84/0	100	98.8	95.2	100	2/18	16/4	2/18
*Enterococcus faecalis*	10/1	94/0	100	98.9	90.9	100	2/8	9/1	2/8
*Staphylococcus saprophyticus*	10/0	94/0	100	100	100	100	0/10	7/3	0/10
Negative result determination									
Total	0/0	30/0		100		100	0/30	7/23	0/30
Urethral microbiota^*d*^	0/0	20/0		100		100	0/20	6/14	0/20
No growth	0/0	10/0		100		100	0/10	1/9	0/10

^*a*^ In total, 104 clinical urine samples were analyzed.

^*b*^ The term “bacteriuria” is used when a significant amount of the five pathogens included in the study was detected.

^*c*^ One sample was placed in double categories (false negative and false positive) since the microorganism identification led to the wrong conclusion.

^*d*^ Urethral microbiota is considered contamination during sampling and is, therefore, clinically negative.

In the second part of the study (prospective), UTI-lizer demonstrated 100% sensitivity and 89.6% specificity in the detection of significant bacteriuria within the intended use of the test (*n* = 121), which covered 88.3% of all samples (*n* = 137). Additionally, 95.5% sensitivity and 87.3% specificity in identifying the five microorganisms with UTI-lizer when compared to results obtained from the microbiology laboratory. It is obvious that the majority of samples was positive for *E. coli*, which is also the local majority pathogen. The results of the retrospective study are summarized in Table 3.

**TABLE 3:** Prospective study results: performance of UTI-lizer in determining bacteriuria and identifying specific microorganisms^*a*^

Identification	No. of true/false positives	No. of true/false negatives	Sensitivity (%)	Specificity (%)	Positive predictive value (%)	Negative predictive value (%)
Bacteriuriadetermination **^*b*^**	44/8	69/0	100	89.6	84.6	100
Microorganism identification **^*c*^**						
Total	42/10	69/2	95.5	87.3	80.8	97.2
Gram-negative bacteria ID						
Total	40/8	73/1	100	91.4	85.1	100
*Escherichia coli*	38/5	78/1	97.4	94	88.4	98.7
*Klebsiella pneumoniae*	2/1	118/0	100	99.2	66.7	100
*Proteus mirabilis*	0/2	119/0		98.4	0	100
Gram-positive bacteria ID						
Total	2/2	116/1	66.7	98.3	50	99.2
*Enterococcus faecalis*	2/2	117/0	100	98.9	90.9	100
*Staphylococcus saprophyticus*	0/0	120/1	0	100		99.2
Negative result determination^*d*^	0/8	69/0		89.6		100

^*a*^ In total, 121 clinical urine samples were analyzed.The results obtained from dipsticks with multi-color (*n* = 11) growth were not interpreted, and no conclusions were drawn from them. Two *Staphylococcus aureus*, one *Citrobacter koseri*, one *Aerococcus urinae*, and one *Streptococcus agalactiae* were excluded as they are not within the intended use of this study.

^*b*^ The term “bacteriuria” is used when a significant amount of the five pathogens included in the study was detected.

^*c*^ Two samples were placed in double categories (false negative and false positive) since the microorganism identification led to the wrong conclusion.

^*d*^ No growth, urethral, and mixed microbiota are considered as negative results.

## DISCUSSION

### Bacteriuria determination with UTI-lizer

The retrospective study results show that the results obtained with the UTI-lizer tests were consistent with standard laboratory testing for the determination and species identification of bacteriuria. On the other hand, the prospective study showed some false positive results (8/121) when using UTI-lizer. It should be noted that tests were not started simultaneously but within the first 10 hours of the laboratory workflow. Therefore, we speculate that contamination during sample inoculation for standard culturing or uncontrolled storage time may have contributed to these additional positive results. Further studies with parallel urine culturing are needed to confirm these conclusions. Despite this, the UTI-lizer showed consistent results with high sensitivity and negative predictive values in determining bacteriuria. Additionally, the small footprint of the test decreases the variation and user dependency during sample inoculation and offers a simple and user-independent readout of the result via digital image processing of the photograph of the device after incubation. These results suggest that UTI-lizer is useful for rapidly ruling out bacterial infections and reducing the unnecessary use of antibiotics, especially when performed in primary care or at the PoC. The test could also help reduce the number of negative urine samples sent to central laboratories, easing the burden of detection of bacteriuria on the healthcare system. It should be noted that the new test is culture based, and that in certain cases, overnight incubation will be the logistically preferred method still.

### Pathogen identification with UTI-lizer

UTI-lizer can successfully detect five main UTI pathogens, but only four can be accurately identified. This is because *S. saprophyticus* growth results in a similar red/pink color change as *E. coli* growth, and since *E. coli* is the more prevalent cause of UTIs, digital analysis of UTI-lizer interpreted red/pink color as *E. coli* growth in the prospective study. This led to one *S*. *saprophyticus* sample being misidentified as *E. coli*. Of note, a simple approach of adding another type of chromogenic media which is selective to Gram-negative bacteria (e.g., eosin methylene blue agar) would solve this problem. Additionally, one *E. coli* sample was categorized as *P. mirabilis* in the prospective study, and one *K. pneumoniae* sample was categorized as *E. faecalis* in the retrospective study. Suggested approach of adding additional selective chromogenic media in the different holes of the same UTI-lizer dipstick would improve the color panel for the identification of species and would solve these issues as the suggested format has ability to do multiplexed testing.

Samples with two *Staphylococcus aureus*, one *Citrobacter koseri*, one *Aerococcus urinae*, and one *S*. *agalactiae* were excluded as they were not within the intended use of this study. Additionally, 11 dipsticks with multi-color growth were not interpreted, and no conclusions were drawn. Therefore, UTI-lizer in its current form showed a coverage rate of 88.3% for all samples (*n* = 137) in the prospective study (Table 3). It should be noted that *S. agalactiae* is not considered a significant pathogen in most cases and is mostly of clinical importance in complicated UTIs (27) or in cases of pregnancy or expected pregnancy. Hence, we cannot conclude on the quality of use for the new test in case of mixed pathogens, and some minority pathogens may remain hard to identify although it is possible to detect the growth in holes with continuous monitoring of dipstick during the incubation. However, it’s important to note that confirming this aspect falls outside the scope of our current study, and additional studies must be conducted to draw such a conclusion.

Overall, UTI-lizer was able to detect 67/68 isolates of *E. coli*, showing that the test has high sensitivity in identifying the most common and important pathogen causing UTIs. Additionally, the 88.3% test coverage rate with satisfactory identification results shows its potential as a PoC tool. However, the variety of bacterial species and the need for confirmatory tests and AST make conventional microbiological urine culture currently hard to replace in clinical microbiology laboratories. Still, UTI-lizer can replace all dipsticks that were of prior use in the testing laboratory (see below). UTI-lizer can be used for all patient categories but those with community-acquired UTI who visit the emergency care department may be a particularly important group of users.

### Use of nitrite and leukocyte esterase test

Using a combined nitrite and esterase test for predicting UTIs was effective in only 36.5% of the samples tested. However, this test was noted to be 100% specific, as no urine samples with negative results were diagnosed as being derived from a patient with UTI. Previous studies have shown that the sensitivity of the nitrite test alone, or in combination with the leukocyte esterase test, varies greatly depending on the bacterial concentration of the urine sample, with lower sensitivity (0%–26%) for bacterial concentrations of 10^3^ CFU/mL (28, 29) and higher sensitivity (53%–84%) for higher concentrations of 10^5^ CFU/mL (28, 30). Although combining the nitrite and leukocyte esterase test provides improved performance (28), these tests should not be relied upon as the sole method for diagnosing UTIs and should always be complemented by other methods, such as urine culture (30). Additionally, the disadvantage of these tests is their inability to identify the specific pathogen causing the UTI. According to the European Association of Urology guideline, urine dipstick tests are rated as “weak” in strength rating for uncomplicated cystitis and are rarely used for complicated UTIs (31). We here claim that the UTI-lizer provides pragmatic progress in this respect.

### Digital analysis of test results

Digital analysis of scanned test images resulted in slightly better performance than naked-eye evaluation performed by a single, well-trained user. The use of cell phone photos as an alternative to scanned images was also explored in this study. Preliminary findings can be found in Supplementary Information 2. The authors believe that digital image analysis could greatly improve the accuracy, reliability, and objectivity of the method when the test is performed by untrained users or in non-standardized settings.

### Conclusion and future outlook

This study shows that UTI-lizer is a viable solution for the detection and specification of bacteriuria as results align with laboratory standards. This demonstrates that UTI-lizer has the potential to optimize and improve UTI diagnosis and management in primary care and PoC settings. The rapid test handling time and lack of complicated equipment needed for conducting the test make UTI-lizer an attractive solution for decentralized bacteriuria screening. Further studies will help to determine their value in improving patient outcomes.

Additionally, the “digitized” culturing format of UTI-lizer allows further multiplexing for extensive bacterial identification and also including AST. A simple strategy of filling certain wells of the dipsticks with various selective agars, chromogenic substrates, or different antibiotics can transfer UTI-lizer into a bacterial detection and identification device, which, compared to traditional culture plates, decreases the demand on central laboratory equipment and facilitates bacterial cultivation and AST at the PoC.

## SUPPLEMENTAL MATERIAL

Supplementary information spectrum.03613-23-s0001.pdf Supplementary Information 1 (MALDI-TOF mass spectroscopy). Supplementary Information 2 (UTI-lizer readout by naked eye and cellphone camera), and Supplementary Information 3 (Definitions).

ASM does not own the copyrights to Supplemental Material that may be linked to, or accessed through, an article. The authors have granted ASM a non-exclusive, world-wide license to publish the Supplemental Material files. Please contact the corresponding author directly for reuse.
