# Supplementary material for: Performance of an innovative culture-based digital dipstick for detection of bacteriuria
Source: Microbiol Spectr. 2023 Dec 13;12(1):e03613-23. doi: 10.1128/spectrum.03613-23 (PMC10783013; doi:10.1128/spectrum.03613-23)
Supplement: Supplementary information — 1 (MALDI-TOF mass spectroscopy). Supplementary Information 2 (UTI-lizer readout by naked eye and cellphone camera), and Supplementary Information 3 (Definitions). [file spectrum.03613-23-s0001.pdf]

SUPPLEMENTARY MATERIALS FOR

"Performance of an innovative culture-based digital dipstick for detection of bacteriuria "

Emre Iseri[1,2], Sara Nilsson[3], Alex van Belkum[4], Wouter van der Wijngaart[1*] and Volkan Özenci[3,5*]

[1]KTH Royal Institute of Technology – Stockholm (Sweden)
[2]UTI-lizer AB – Stockholm (Sweden)
[3]Karolinska University Hospital – Stockholm (Sweden)
[4]BaseClear BV – Leiden (Netherlands)
[5]Karolinska Institutet – Stockholm (Sweden)

SUPPLEMENTARY INFORMATION 1

MALDI-TOF mass spectroscopy for bacteria identification

MALDI-TOF MS was performed according to the guidelines in MIK-document 4447-7 (2019), Karolinska University Hospital, Clinical Microbiology, Huddinge. A bacterial colony was spread on a single position on MBT Biotarget 96 (Bruker Daltonics GmbH, Bremen, Germany). 2.5 mg Alpha-cyano-4-hydroxycinnamic acid (HCCA) matrix (Bruker Daltonics GmbH, Bremen, Germany) was dissolved in organic stem solution (25% acetonitrile, 2% trifluoroacetic acid; Sigma-Aldrich, Munich, Germany). HCAA matrix was applied to all positions containing biomaterial and left to air-dry. Samples were analyzed on Microflex Maldi TOF MS with associated software (Bruker Daltonics GmbH, Bremen, Germany). Spectra were compared in Maldi Biotyper (MBT) Compass Explorer against Bruker's database of bacterial species.

SUPPLEMENTARY INFORMATION 2

<u>Readout of UTI-lizer™ by the naked eye:</u>
Digital analysis of scanned test images showed slightly better performance than naked-eye evaluation in terms of bacteria identification. More specifically, there was only one sample misinterpreted by the naked eye when it is compared to the digital analysis of scanned images. This specific sample containing *E. coli* is categorized as *P. mirabilis* by the user, shown in Supplementary Fig. 1. It should be noted that this part of the study was performed by a single, well-trained user. When untrained users or in non-standardized settings are taken into consideration, authors believe that digital image analysis could greatly improve the accuracy, reliability, and objectivity of the method.

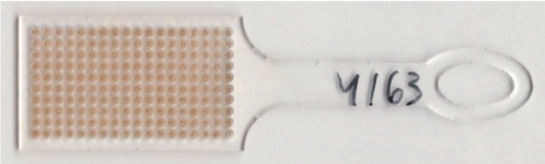

Supplementary Fig. 1 Scanned image of UTI-lizer™ containing *E. coli* categorized as *P. mirabilis* by the user where digital analysis system made correct interpretation.

<u>Readout of UTI-lizer™ by cell phone camera:</u>
The use of cell phone photos as an alternative to scanned images was also explored in this study. Preliminary results show that images taken by iPhone 8S can be used for digital analysis. However, some images required additional correction for differences in light settings, which is not automatized in the current analysis system. Additionally, a limited number of samples in the prospective study have images taken by cellphone camera. Therefore, the findings obtained from cell phone photos were not included in this study. Two examples of readout photos taken under different background lights are shown in Supplementary Fig.2 to give an example. Images are taken on different days with different background lights. They are placed on the same cardboard which is used to do colour correction and white balancing to obtain correct results from these pictures. Images clearly show the difference in colouring due to different background lights.

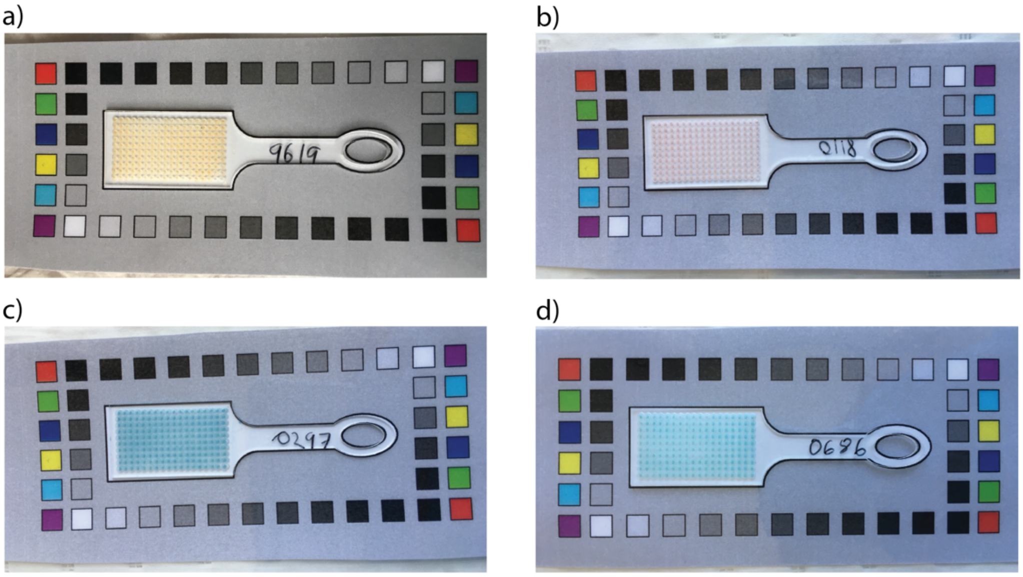

Supplementary Fig. 2 Photographs of UTI-lizer™ taken under various background lights. a) UTI-lizer™ with *P. mirabilis* growth. b) UTI-lizer™ with *E. coli* growth. c) UTI-lizer™ with *K. pneumoniae* growth. d) UTI-lizer™ with *E. faecalis* growth.

SUPPLEMENTARY INFORMATION 3

Predominantly monomicrobial urine samples:
For a urine sample to be considered monomicrobial, any microorganism other than the predominant one must have a concentration below 1/10th of that of the five analysed bacterial species in question.
Urine samples with urethral microbiota:
Urethral microbiota was defined as normal bacterial flora in women, including but not limited to Lactobacillus species and anaerobic bacterial species. It can also refer to a less than 10^4 CFU/mL growth of gram-positive bacteria that does not constitute a pure S. saprophyticus culture.
Urine samples with no growth:
No growth (negative samples) was defined as less than $2x10^2$ CFU/mL of primary pathogens or less than 10^3 CFU/mL of secondary pathogens.
